# The vesicular nucleotide transporter (VNUT) is involved in the extracellular ATP effect on neuronal differentiation

**DOI:** 10.1007/s11302-015-9449-4

**Published:** 2015-04-07

**Authors:** Aida Menéndez-Méndez, Juan Ignacio Díaz-Hernández, M. Teresa Miras-Portugal

**Affiliations:** Facultad de Veterinaria, Departamento de Bioquímica y Biología Molecular IV, Universidad Complutense de Madrid, Madrid, Spain

**Keywords:** VNUT, Neuritogenesis, Retinoic acid differentiation, ATP, Neuroblastoma cells, SLC17A9

## Abstract

Before being released, nucleotides are stored in secretory vesicles through the vesicular nucleotide transporter (VNUT). Once released, extracellular ATP participates in neuronal differentiation processes. Thus, the expression of a functional VNUT could be an additional component of the purinergic system which regulates neuronal differentiation and axonal elongation. In vitro expression of VNUT decreases neuritogenesis in N2a cells differentiated by retinoic acid treatment, whereas silencing of VNUT expression increases the number and length of neurites in these cells. These results highlight the role of VNUT in the neuritogenic process because this transporter regulates the ATP content in neurosecretory vesicles.

**Electronic supplementary material:**

The online version of this article (doi:10.1007/s11302-015-9449-4) contains supplementary material, which is available to authorized users.

## Introduction

Neurotransmission and neuroendocrine signaling depends on the regulated release of a large variety of vesicular-stored signaling molecules. Non-peptidergic compounds require specific vesicular transporters, all of them are members of the solute carrier family (SLC) [1, 2]. Nowadays, it is widely accepted that a single secretory vesicle contains more than one neurotransmitter and requires the presence of the corresponding specific transporters in the membrane vesicles. ATP and a large variety of nucleotidic compounds are among the most frequent substances co-stored with the classical neurotransmitters earlier discovered, such as acetylcoline, catecholamines, serotonine, and even glutamate, or other aminoacidic compounds [3, 4]. To explain the complexity of signaling events upon vesicular release, G. Burnstock coined the term of co-transmission [5].

The abundant presence and distribution of secretory vesicles containing ATP and other nucleotides require the understanding of their transport and storage [6]. Regarding this, some previous work from our group contributed to the kinetic characterization of the vesicular nucleotide transporter (VNUT), showing its dependence on the pH gradient between vesicles lumen and cytosol, its prominent mnemonic behavior and the broad substrate specificity [7] . Recently, Moriyama’s group cloned and identified VNUT as the product of the SLC17A9 gene [8]. Proteoliposomes reconstituted with the purified recombinant protein exhibit ∆ψ-driven and Cl^−^ dependent ATP transport. Expression in secretory tissues such as adrenal gland and suppression by small interfering RNA, together with the concomitant reduction on ATP release, confirmed the SLC17A9 as the VNUT transporter.

Once released, ATP and the other stored nucleotides act on their specific P2X ionotropic or P2Y metabotropic receptor families, located in the same or neighboring cells [9, 10]. A large number of ectonucleotidases accomplish the enzymatic degradation of nucleotides to adenosine and inorganic phosphate, finishing their action on P2 receptors [11–13]. All the molecules with a role in these cellular processes are grouped under the name of purinome, and they are specific for different cell types and physiopathological situations, which require a convenient experimental model. Among these models, embryonic hippocampal neurons and neuroblastoma N2a cell line, where P2X7 receptors are functional and mainly located at the axonal growth cone, have been proved to be suitable to understand the sequence of purinergic events for axonal growth and branching [14–16]. The presence of extracellular ATP and its effect on P2X7 receptors reduces axonal growth through a signaling cascade requiring intracellular Ca^2+^ increase [17, 14]. In contrast, either degradation of ATP by ectonucleotidases activity or antagonizing or silencing of P2X7 receptors is able to increase axonal length [17]. However, the involvement of VNUT in the regulation of neural differentiation and axonal elongation remains unknown. In this report, we have analyzed the consequence of VNUT expression/silencing in the process of neuritogenesis that occurs in N2a cells after treatment with retinoic acid. Thus, VNUT expression decreases the number and length of neurites, and this effect is reversed by the silencing of VNUT expression. These results indicate that the extracellular presence of ATP is linked to its vesicular content and availability to be released, which directly involves the vesicular transporter VNUT in the neuritogenesis and differentiation processes.

## Materials and methods

### Plasmid constructs and design of shRNAs

Mouse VNUT (NM_183161) was amplified from IMAGE consortium bacterial clone (IMAGE clone 4986674) by PCR and ligated into the pcDNA3.1 (+)/myc-His A vector (Life Technologies) generating VNUT flagged with c-myc epitope (VNUT-myc). pRFP-C-RS vector obtained from Origene was used as transfection control. VNUT knockdown was achieved by RNA interference using a vector-based small hairpin RNAs (shRNA) approach (pSUPER.neo.GFP, Oligoengine). Several shRNA targets for VNUT were designed according to a previously reported rational-design protocol [18] and sequence 5′-GAACAAGAAGGAGGCTGGTATCGTGCTCA-3′ was selected. Specificity of the sequence was confirmed by a BLAST analysis for mouse VNUT. As a control, we used the firefly-luciferase-targeted oligonucleotide 5′-CTGACGCGGAATACTTCGA-3′. Synthetic forward and reverse 64-nucleotide oligonucleotides (Sigma-Aldrich) were designed, annealed, and inserted into *Bgl*II-*Hin*dIII sites of the pSUPER.neo.GFP vector following the manufacturer’s instructions. These plasmid constructs express shRNA targeted against VNUT (shVNUT) or luciferase (non-targeting, shNT) transcripts. The concomitant expression of GFP from this vector allowed transfected cells to be identified by fluorescence.

### Cell culture

N2a cells were cultured in Dulbecco’s modified eagle medium (DMEM, Sigma-Aldrich) supplemented with Glutamax® (Invitrogen), penicillin/streptomycin (Invitrogen), and 10 % heat-inactivated FBS (Gibco®, Life Technologies). Cells were grown at 37 °C in a humidified atmosphere containing 5 % CO_2_. For differentiation studies, cells were seeded at a density of 10^5^ cells/cm^2^ in low-serum medium (DMEM containing 0.5 % FBS) supplemented with 10 μM retinoic acid (Sigma-Aldrich), renewed every 2 days as described by Tremblay et al. (2010) [19]. The assays were performed on day five of differentiation.

### Transfections

N2a cells were plated at 8 × 10^5^ cell/cm^2^ in 6-well plates and transiently transfected with 2.5 μg of the different plasmids using Lipofectamine™ 2000 (Life Technologies) following the manufacturer’s instructions. After 6 h, the medium was removed and the cells were further incubated for the indicated time periods in DMEM supplemented with Glutamax® (Invitrogen), penicillin/streptomycin (Invitrogen), and 10 % heat-inactivated FBS. In the case of co-transfections with two plasmids, 1.25 μg was used for each plasmid in order not to exceed total DNA quantity.

### RT-PCR and quantitative real-time PCR

Total RNA was purified from cultured N2a cells using a SpeedTools Total RNA Extraction kit (Biotools) according to the manufacturer’s instructions. After digestion with TURBO DNase (Ambion), 1 μg of total RNA was quantified and reversed transcribed using M-MLV reverse transcriptase, 6 μg of random primers, and 350 μM dNTPs (all from Invitrogen). The quantitative real-time PCR (qPCR) reaction were carried out using LuminoCt qPCR ReadyMix (Sigma-Aldrich), 5 μl of the cDNA previously synthesized and 1.25 μl of specific commercial oligonucleotide primers for mouse VNUT (TaqMan® Gene Expression Assay, Life Technologies Assay ID Mm00805914_m1), as well as for the housekeeping gene glyceraldehyde 3-phosphate dehydrogenase (GAPDH, TaqMan® Gene Expression Assay, Life TechnologiesAssay ID Mm99999915_g1) in 25 μl final volume. The reaction was realized in a StepOnePlus Real-Time PCR System (Applied Biosystems) as follows: denaturation, one cycle of 95 °C for 20 s, followed by 40 cycles each of 95 °C for 1 s and 60 °C for 20 s. The results were normalized as indicated by parallel amplification of the endogenous housekeeping gene GAPDH.

### Western blotting

N2a cells were lysed and homogenized for 1 h at 4 °C in lysis buffer containing 50 mM Tris*/*HCl, 150 mM NaCl, 1 % Nonidet P40 and Complete^TM^ Protease Inhibitor Cocktail Tablets (Roche Diagnostics GmbH), pH 7.4. Separation of the proteins was performed on 10 % SDS-PAGE gels. Proteins were transferred to nitrocellulose membranes, blocked for 1 h at room temperature (RT) with 5 % nonfat dried milk in tween-phosphate-buffered saline (137 mM NaCl, 2.7 mM KCl, 5 mM Na_2_HPO_4_-7H_2_0, 1.4 mM KH_2_PO_4_, and 0.1 % Tween; pH 7.4) (PBS-T), and incubated overnight at 4 °C with anti-c-myc antibody (Invitrogen) at 1:2500 or anti-α-tubulin antibody (Sigma-Aldrich) at 1:10,000. Blots were then washed in PBS-T, and incubated for 1 h at RT with goat anti-mouse IgGs coupled to horseradish peroxidase (Dako), at 1:5000 dilution. Protein bands were detected by ECL chemiluminescence (Amersham GE Healthcare). The expression of VNUT was standardized by the expression of α-tubulin of the same experiment. Images were captured with ImageQuant LAS 500 (GE Healthcare Life Sciences) and analyzed using ImageQuant TL.

### ATP release measurement

ATP release was measured using ENLITEN® rLuciferase/Luciferin reagent (Promega). Culture medium (100 μl) was collected under various experimental conditions and centrifuged at 600 × *g* for 5 min at 4 °C, and 10 μl aliquots of supernatant were transferred to wells of a 96-well plate placed on ice. Before the start of the experiments, N2a cells were bathed in Mg^2+^-free Locke’s buffer supplemented with 100 μM ARL 67156, a competitive inhibitor of ecto-ATPases [20] for 30 min at 37 °C and the medium was collected to measure basal ATP. Then, cells were stimulated by adding ionomycin (2 μM final concentration). Five minutes later, extracellular medium was again collected to measure evoked ATP concentration. The 96-well plate was set in a FLUOstar OPTIMA Microplate Luminometer (BMG LABTECH GmbH), and 100 μl of rLuciferase/Luciferin reagent was automatically injected into each well at RT (25 °C).

### Immunocytochemistry

N2a-transfected cells cultured on coverslips placed in 35-mm dishes were fixed with 4 % PFA for 15 min and rinsed with PBS twice for 10 min. Afterwards, cells on coverslips were permeabilized in blocking solution (Triton X-100 0.1 %, FBS 5 %, and BSA 10 % in PBS) for 1 h at RT. This was followed by incubation with primary antibodies: anti-c-myc (1:200), anti-β-III tubulin (1:1000) (Promega) and anti-synaptophysin (1:200) (Synaptic Systems). Subsequently, cells were washed with PBS three times and incubated for 1 h at RT with Alexa Fluor 594® donkey anti-rabbit, Alexa Fluor 488® goat anti-mouse, and Alexa Fluor 647® goat anti-mouse (all from Invitrogen). Nuclei were counterstained with 4′,6-diamidino-2-phenylindole (DAPI, Invitrogen), a fluorescent stain that binds strongly to DNA. Coverslips were mounted on glass slides using FluoroSave^TM^ Reagent (Calbiochem). Images were acquired using a confocal laser microscope (Leica TCS SPE) using 20× W/IR objective lenses. The analysis of neurite length and ramifications was carried out using Image J v1.49j (NIH) and Neuron J plugin v1.4.2 [21]. Neurite outgrowth was quantified by determining the percentage of cells that present neurites once or twice longer than the soma diameter (SD) as described by Ravichandra and Joshi (1999) [22]. Ten different fields in the culture dishes containing 70 cells in each field were randomly selected and counted. The mean percentage of neurite-bearing cells was obtained for each culture. Each assay was repeated six times in three independent cultures.

### Statistical analysis

Data are shown as mean values ± standard error of the mean (SEM). All experiments shown were reproduced 3–6 independent times. Figures and statistical analyses were generated using GraphPad Prism 6 (GraphPad Software). Results were analyzed by unpaired Student’s *t* test or ANOVA with Tukey or Sidak’s multiple comparisons test as indicated. Neurite length was compared using Kolmogorov-Smirnov analysis on the cumulative frequency distribution. The statistical test used and *P* values are both indicated in each figure legend. *P* ≤ 0.05 was considered statistically significant.

## Results

### Design of molecular tools for VNUT expression

**Fig. 1:**
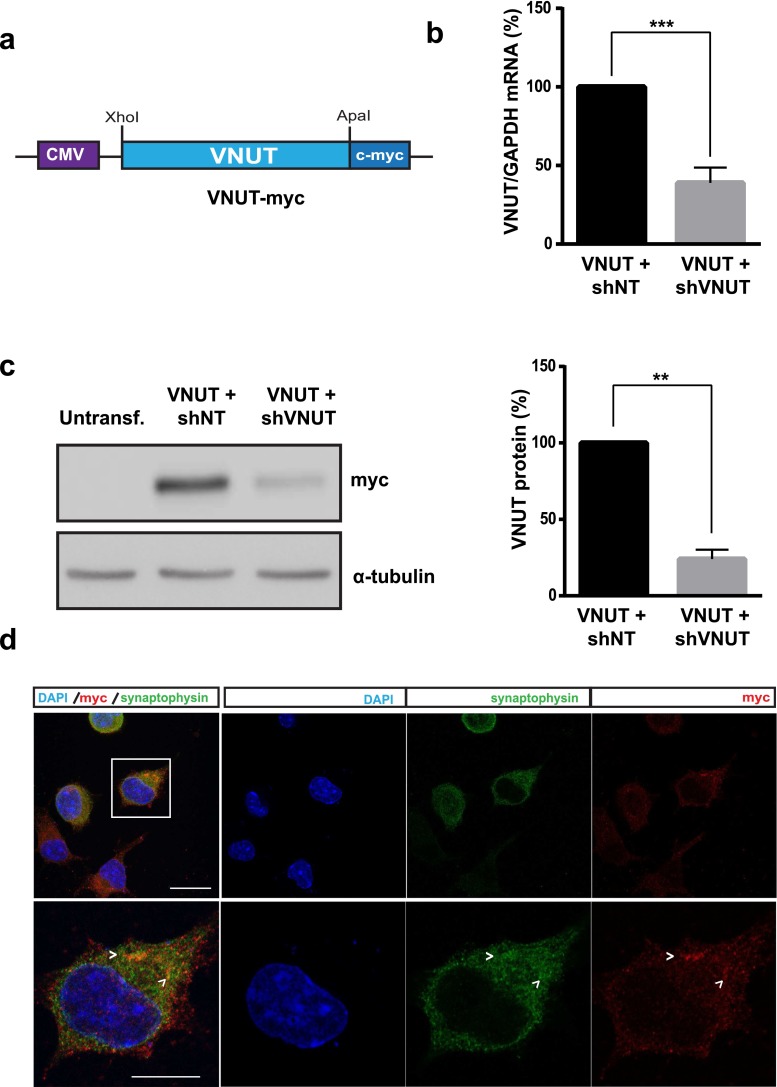
Characterization of molecular tools to study expression or silencing of VNUT expression in N2a cells. **a** Schematic representation of the construct used for expression of VNUT in N2a cells. **b** VNUT mRNA levels analyzed by qPCR of transfected N2 cells with VNUT-myc and either shNT or shVNUT. shNT was used as negative control. The values represent the mean ± SEM (*n* = 3, ***P < 0.001, unpaired Student’s *t* test). **c** Western blotting of untransfected N2a cells or N2a cells transfected with either VNUT-myc and shNT or shVNUT. α-Tubulin was used as internal loading control. *Graph bars* represent the mean ± SEM (*n* = 3, **P < 0.01, unpaired Student’s *t* test). **d** Representative confocal images showing immunostaining against c-myc epitope fused to VNUT (*red*) and synaptophysin (*green*) of N2a cells transfected with VNUT-myc. Nuclei are counterstained with DAPI (*blue*). *Scale bar* 10 μm. *Inset* represents 4× magnification of indicated cell. *Arrows* indicate the most prominent VNUT and synaptophysin positive vesicles. 5 μm

In order to validate the molecular tools used in this study, N2a cells were transfected with the designed constructs VNUT-myc (Fig. 1a) and shRNAs either control (shNT) or specifically designed against VNUT (shVNUT). After 24 h, the transfection efficiency was about 85 % and mRNA and protein levels of VNUT were analyzed by qPCR (Fig. 1b) and Western blotting (Fig. 1c), respectively. These experiments demonstrated that VNUT is successfully expressed in transfected N2a cells. Moreover, the expression of this transporter was reduced when N2a cells were co-transfected with VNUT-myc and shVNUT (Fig. 1b, c). Likewise, immunocytochemistry assays were performed incubating transfected N2a cells with anti-c-myc antibody, and a vesicular co-localization with vesicular marker synaptophysin could be observed in confocal fluorescence images (Fig. 1d).

### Retinoic acid-induced N2a differentiation keeps VNUT overexpression in transfected cells

**Fig. 2:**
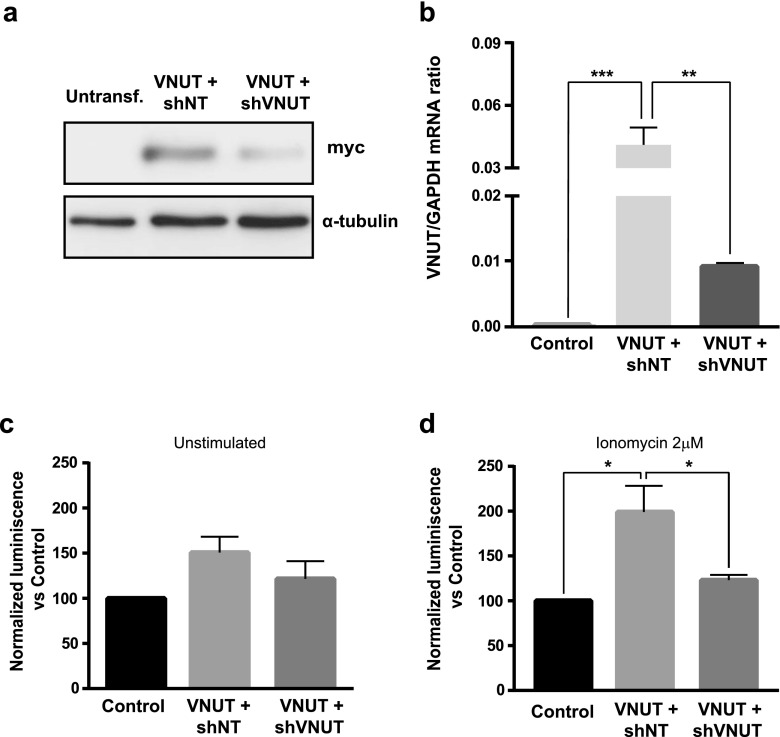
Expression of a functional VNUT in N2a cells after differentiation with retinoic acid. **a** Western blotting of untransfected or transfected differentiated N2a cells with VNUT-myc after a 5-day differentiation process with retinoic acid (10 μM). **b** qPCR of VNUT mRNA levels of differentiated N2a cells transfected with different plasmids. N2a cells were transfected with control plasmid, VNUT-myc + shNT or VNUT-myc + shVNUT as indicated. The values represent the mean ± SEM (*n* = 3, **P < 0.01, ***P < 0.001, ANOVA with the Tukey’s multiple comparisons test) normalized by the content of GAPDH transcript. **c** Luminescence activity of ATP release to the extracellular medium by transfected N2a cells without any stimulation. Luminescence was normalized against control cells. The values represent the mean ± SEM (*n* = 3, non-significant, ANOVA with the Tukey’s multiple comparisons test). **d** Luminescence activity of ATP release to the extracellular medium by transfected N2a cells after stimulation with Ca^2+^ ionophore ionomycin (2 μM). Luminescence was normalized against control cells. The values represent the mean ± SEM (*n* = 3, **P < 0.01, ANOVA with the Tukey’s multiple comparisons test)

Transfected N2a cells were submitted to retinoic acid differentiation according to Tremblay et al. (2010) [19]. Western blotting and qPCR assays were performed to confirm that expression of VNUT is maintained in differentiated N2a cells. After 5 days with retinoic acid, VNUT mRNA and protein levels were analyzed. The expression of the transporter was detected in differentiated N2a cells co-transfected with VNUT and shNT by Western blotting (Fig. 2a) and qPCR (Fig. 2b) assays. The reduction on the expression of VNUT after 5 days of differentiation, compared with cells freshly transfected, was caused by the loss of transfected cells associated to transient transfections, where only about 15 % of cells remain transfected. On the other hand, VNUT mRNA levels were reduced in differentiated N2a cells co-transfected with VNUT and shVNUT (Fig. 2a, b). Furthermore, the functionality of the vesicular transporter was confirmed by using the luciferin-luciferase assay to quantify the luminescence signal produced by the released ATP (Fig. 2c). Previous studies demonstrated that N2a cells have a mechanism of ATP release depending on the increase in intracellular [Ca^2+^], involving the exocytotic machinery [16]. Differentiated N2a cells were stimulated with Ca^2+^-selective ionophore, ionomycin 2 μM, for 5 min and extracellular medium was collected with or without stimulation. Cells co-transfected with VNUT and shNT showed the highest values of luminescence, which decreased when co-transfected with VNUT and shVNUT (Fig. 2c, d). These results indicate that the vesicular nucleotide transporter is functional when it was expressed in differentiated N2a cells and kept functional during retinoic acid differentiation process.

### Neuritogenesis is reduced in N2a overexpressing VNUT

**Fig. 3:**
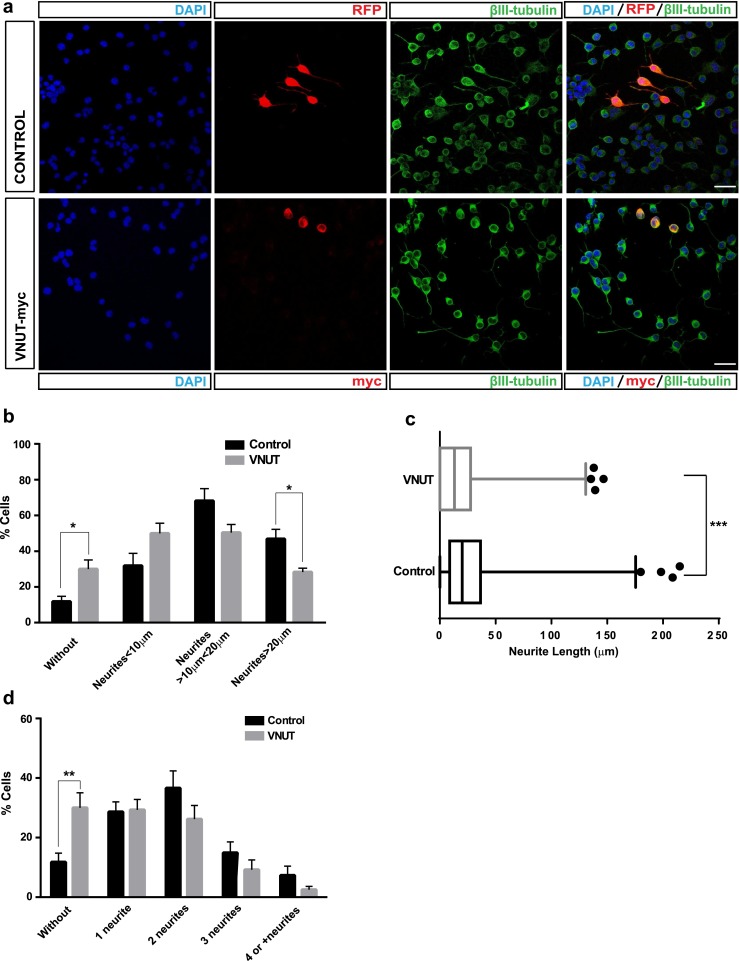
Expression of VNUT decreases neuritogenesis in differentiated N2a cells. **a** Representative confocal images of transfected N2a cells with control (RFP) (*upper panels*) or VNUT-myc (*lower panels*). Transfected control cells were identified by RFP fluorescence and cells expressing VNUT by anti-c-myc antibody (*red*). N2a cells were immunostained with anti-β-III tubulin antibody (*green*) for the morphological analysis. Nuclei were counterstained with DAPI. *Scale bar*: 25 μm. **b** Percentage of transfected N2a cells without neurites or with neurite length lower than soma diameter (<SD, 10 μm), or higher than once SD (>1SD, 10 μm) or twice SD (>2SD, 20 μm). N2a transfected with control RFP are represented by *black bars* and N2a transfected with VNUT by *gray bars* (*n* = 6, *P < 0.05, **P < 0.01, ANOVA with Sidak’s multiple comparisons test). **c** Boxplot representation of the main neurite length in micrometer. Wiskers contains 10–90 % of total measures (***P > 0.001 with Kolmogorov-Smirnov analysis on the cumulative frequency). **d** Percentage of transfected N2a cells with different number of neurites. N2a transfected with RFP are represented by *black bars* and N2a transfected with VNUT by *gray bars*. Data show the mean ± SEM of 70 cells (*n* = 6, *P < 0.05, **P < 0.01, ANOVA with Sidak’s multiple comparisons test)

After verification that VNUT is functional and its expression remains in differentiated N2a cells, we wanted to analyze the possible effects that expression of VNUT could produce in the neuritogenesis of these cells. To study the probable morphological changes, N2a cells were transfected either with control or VNUT-myc construct, and identified by immunostaining with anti-c-myc antibody (for VNUT-myc-transfected cells) or RFP fluorescence from pRFP-C-RS vector as transfection control (Fig. 3a). This vector has a red fluorescent protein (RFP)-coding sequence which allows to identify transfected cells (Fig. 3a). A specific anti-β-III tubulin antibody was used to observe the neuronal morphology (Fig. 3a) and go ahead with the quantification of neurites.

In N2a cells that expressed VNUT the percentage of cells without neurites was significantly increased compared to control (30.0 % ± 5.0 versus 11.8 % ± 2.9) (Fig. 3b and d). Similarly, the expression of VNUT decreased the median of length of the principal neurite 13.07 μm for VNUT transfected cells compared with 20.00 μm for control cells (Fig. 3c) (*P* < 0.0001 with Kolmogorov-Smirnov analysis on the cumulative frequency distribution). In addition, if a distribution of cells is made accordingly to neurite length, the expression of VNUT increases the percentage of cells with neurites shorter than the soma diameter (SD 10 μm) (49.9 ± 5.7 % versus 31.9 ± 6.9 %) (Fig. 3b). Otherwise, the percentage of cells with neurites longer than SD (>10 μm) (50.4 ± 4.5 % versus 68.1 ± 6.9 %) and neurites twice longer than SD (>20 μm) (28.3 ± 2.2 % versus 46.8 ± 5.5 %) decreased in those cells that expressed VNUT (Fig. 3b). In the same way, expression of VNUT decreased the percentage of cells with two (26.2 ± 4.5 % versus 36.6 ± 5.8 %), three (9.2 ± 3.3 % versus 14.9 ± 3.9 %), and four or more (2.5 ± 1.1 % versus 7.3 ± 3.1 %) neurites compared to control cells (Fig. 3c).

### VNUT knockdown restores differentiation process in N2a cell line

**Fig. 4:**
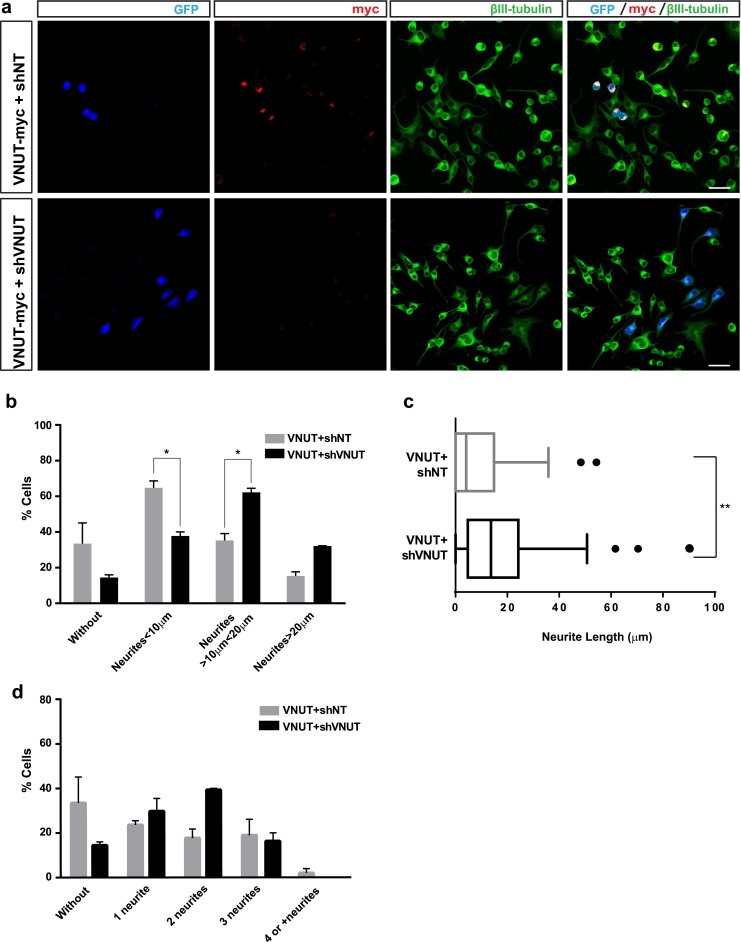
Knockdown of VNUT expression increases neuritogenesis in differentiated N2a cells. **a** Representative confocal images of N2a cells co-transfected with VNUT-myc and either shNT (*upper panels*) or shVNUT (*lower panels*). Transfected cells were identified by GFP fluorescence (*blue*). N2a cells were immunostained with anti-c-myc (*red*) and anti-β-III tubulin (*green*) antibodies. *Scale bar*: 25 μm. **b** Percentage of transfected N2a cells without or with neurite length lower than soma diameter (<SD, 10 μm) or higher than once SD (>1SD, 10 μm) or twice SD (>2SD. 20 μm). N2a transfected with VNUT^+^ shNT are represented by *black bars* and N2a transfected with VNUT^+^ shVNUT by *gray bars* (*n* = 6, *P < 0.05, **P < 0.01, ANOVA with Sidak’s multiple comparisons test). **c** Boxplot representation of the main neurite length in micrometer. Wiskers contains 10–90 % of total measures (**P > 0.01 with Kolmogorov-Smirnov analysis on the cumulative frequency). **d** Percentage of transfected N2a cells with different number of neurites. N2a transfected with VNUT^+^ shNT are represented by *black bars* and N2a transfected with VNUT^+^ shVNUT by *gray bars*. Data show the mean ± SEM of 70 cells (*n* = 6, *P < 0.05, **P < 0.01, ANOVA with Sidak’s multiple comparisons test)

In order to corroborate that the observed effect in the differentiation of N2a cells is due to expression of the vesicular transporter, shVNUT was used to knockdown its expression. N2a cells were co-transfected with VNUT-myc and either shNT (control) or shVNUT. Cells co-transfected with both plasmids were identified by shRNA-GFP fluorescence emission and anti-myc immunolabeling addressed to detect VNUT-myc (Fig. 4a). The knockdown of VNUT increased the median of length of the principal neurite, from 9.1 μm for VNUTand shNT cells to 18.3 μm for VNUT and shVNUT transfected cells (Fig. 4c) (*P* < 0.001 with Kolmogorov-Smirnov analysis on the cumulative frequency distribution). The knockdown of VNUT expression decreased the percentage of cells without neurites (33.4 ± 11.7 % to 14.5 ± 1.5 %) (Fig. 4b). Likewise, the same effect was observed in cells with neurites shorter than SD (<10 μm) whose percentage significantly decreased (64.7 ± 3.9 % to 37.7 ± 2.3 %) (Fig. 4c). However, the percentages of cells with both neurites longer than SD (>10 μm) (35.2 ± 3.8 % to 62.3 ± 2.3 %) (Fig. 4c) and neurites twice as long as SD (>20 μm) (15.3 ± 2.3 % to 32.1 ± 0.1 %) were increased compared to N2a cells co-transfected with VNUT and shNT. Regarding the number of neurites, the effect of VNUT knockdown was not as evident as in the observed neurite outgrowth (Fig. 4d). These results indicate that the reduction of VNUT expression increases neuritogenesis in differentiated N2a cells.

## Discussion

The aim of this experimental work was to demonstrate that the vesicular nucleotide transporter (VNUT) is a key component required to accomplish the cellular biological events involving extracellular ATP in neurosecretory cells. Nucleotide P2X and P2Y receptors are present in most mammalian cells, and their physiological or pathological signaling are unveiled step by step [9, 23, 24]. For secretory cells, the cloning and characterization of the elusive VNUT opened new possibilities of understanding the ATP extracellular cycle and its relation with secretory tissues-related diseases [8, 25–27]. The first tissues studied were from neural or neuroendocrine origin, and the presence of VNUT was demonstrated by immunohistochemistry and RNA in situ hybridization [28–30]. It is worth emphasizing that synaptic vesicles and chromaffin cell granules are able to store ATP and other nucleotides up to 0.1 M, among them are the diadenosine polyphosphates which are agonists on several P2X and P2Y receptors [31].

**Fig. 5:**
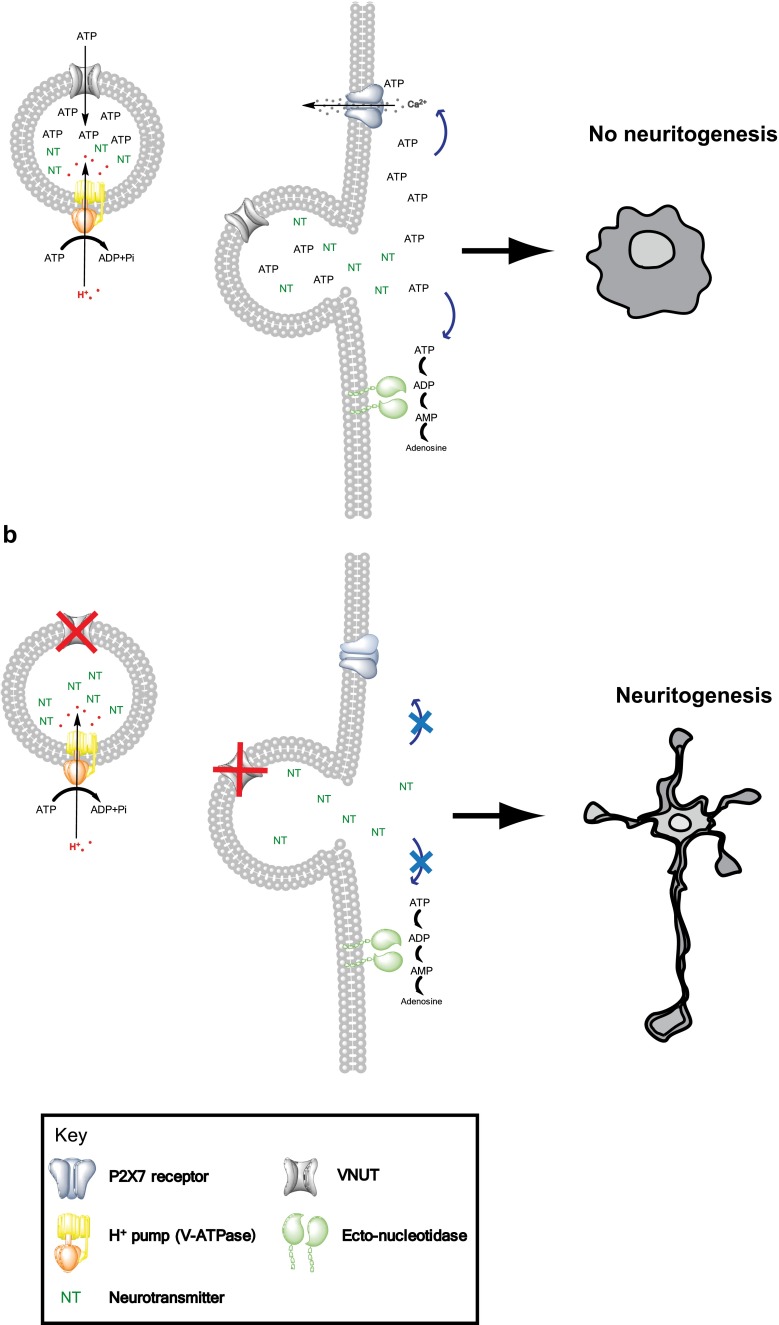
VNUT is a component of the purinergic machinery required for control of axonal growth and neuritogenesis. ATP is stored in secretory vesicles through VNUT; this transporter uses the generated gradient (Δ μ_H+_) by V-ATPase to translocate ATP into vesicles. ATP is released to extracellular medium through calcium-dependent mechanism. Once released, ATP can activate purinergic receptors, such as P2X7, in an autocrine manner that results in various physiological cellular events. Simultaneously, ATP can be degraded to its metabolites by ectonucleotidases activity. **a** The abundant presence of extracellular ATP induces P2X7 activation, and the intracellular Ca^2+^ increases results on axonal length and neuritogenesis reduction in this cellular model [16]. **b** On the contrary, a decrease or destruction of extracellular ATP results in an increase of axonal length and branching

Neuroblastoma N2a cell line has proved to be a valuable model to analyze cell biology events and consequently selected to understand the role of VNUT in neural differentiation events [14, 15]. A simplified scheme of this process is shown in Fig. 5. On the other hand, these cells express very small amounts of endogenous VNUT, which was a requisite for better understanding of the expression effects [16]. Expression of the VNUT in secretory cells has already been reported in PC12 cells, a chromaffin cell model, being able to increase ATP secretion in depolarizing conditions [8]. In our model, expression of VNUT co-localizes with the vesicular marker synaptophysin, and as reported for PC12 cell line, the membrane depolarization increases the ATP release. These results are important experimental facts to consider VNUT as a functional transporter. Furthermore, sustained expression of VNUT is observed when N2a cells are subjected to retinoic acid differentiation, this being a necessary condition to evaluate the ATP effects on neuritogenesis and differentiation.

In this regard, expression of VNUT clearly reduces the neuritogenic processes in N2a cells compared to control cells. Changes induced in the cellular morphology allow a precise quantification and comparison of length of neurite processes in VNUT expressing and non-expressing cells. Additional experiments to corroborate the VNUT negative effect on neuritogenic processes required N2a cells co-transfected with VNUT and a knockdown, shVNUT. These cells, where VNUT expression was reduced, recovered their morphology with more prominent neuritogenic processes. This confirms the hypothesis of VNUT is involved in neuronal differentiation as part of a mechanism requiring extracellular ATP acting through P2X7 receptor at the axonal growth cone [32, 14, 17]. The physiological relevance and future perspectives of VNUT as a pharmacological target derive from its capacity to increase extracellular ATP levels. ATP vesicular release has also been reported from the mucin granules of airway epithelial cells, microglia, and a large variety of neuroendocrine secretory tissues [33, 25, 30]. The physiopathological role of extracellular ATP and its exocytotic release in many tissues supports VNUT as a new and relevant pharmacological target.

## Electronic supplementary material

Supplementary figure 1 N2a expression of P2 receptors is not affected by the expression of VNUT. a) P2X7 mRNA levels analyzed by qPCR of untransfected N2 cells and transfected with RFP as control and VNUT-myc. The values represent the mean ± SEM (*n* = 3, non -significant, unpaired Student’s *t*-test). b) P2Y2 mRNA levels analyzed by qPCR of untransfected N2 cells and transfected with RFP as control and VNUT-myc. The values represent the mean ± SEM (*n* = 3, non -significant, unpaired Student’s *t*-test). (c) Western Blotting against P2X7 and P2Y2 of untransfected N2a cells or N2a cells transfected with either VNUT-myc or RFP as control. α-tubulin was used as internal loading control. d-e) Histogram of measured proteins levels of P2X7 (d) and P2Y2 (e) represents normalized values with non-transfected control of the mean ± SEM (*n* = 3, non-significant, unpaired Student’s *t* test). (GIF 52 kb)

High resolution image (EPS 1606 kb)

## Abbreviations

VNUT: Vesicular nucleotide transporter
RFP: Red fluorescent protein
GFP: Green fluorescent protein
N2a: Neuro-2a
shRNA: Small hairpin RNA
