# Supplementary figures and images for: The vesicular nucleotide transporter (VNUT) is involved in the extracellular ATP effect on neuronal differentiation
Source: Purinergic Signal. 2015 Apr 7;11(2):239–49. doi: 10.1007/s11302-015-9449-4 (PMC4425722; doi:10.1007/s11302-015-9449-4)

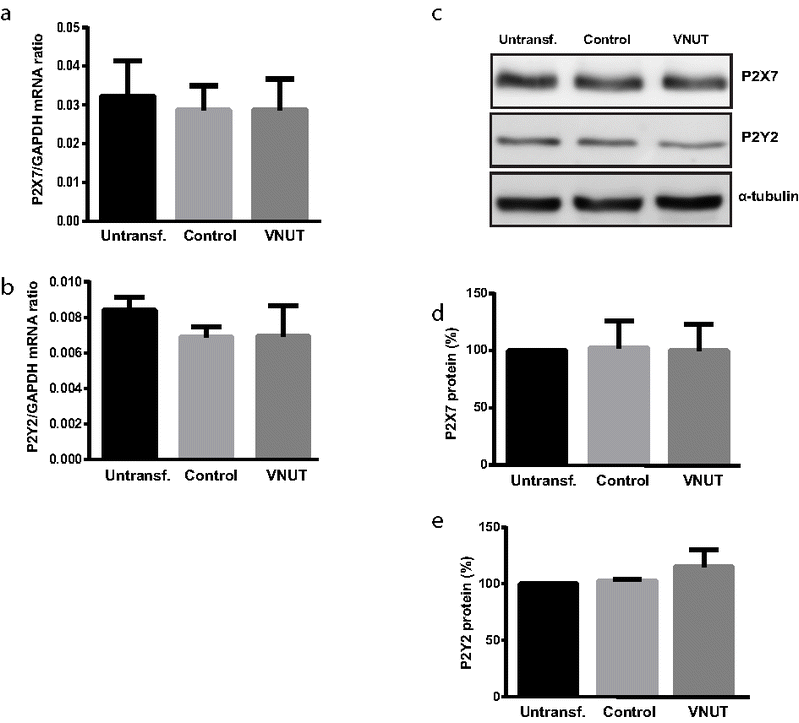

Supplement: Supplementary file 1 — N2a expression of P2 receptors is not affected by the expression of VNUT. a) P2X7 mRNA levels analyzed by qPCR of untransfected N2 cells and transfected with RFP as control and VNUT-myc. The values represent the mean ± SEM (n = 3, non -significant, unpaired Student’s t-test). b) P2Y2 mRNA levels analyzed by qPCR of untransfected N2 cells and transfected with RFP as control and VNUT-myc. The values represent the mean ± SEM (n = 3, non -significant, unpaired Student’s t-test). (c) Western Blotting against P2X7 and P2Y2 of untransfected N2a cells or N2a cells transfected with either VNUT-myc or RFP as control. α-tubulin was used as internal loading control. d-e) Histogram of measured proteins levels of P2X7 (d) and P2Y2 (e) represents normalized values with non-transfected control of the mean ± SEM (n = 3, non-significant, unpaired Student’s t test). (GIF 52 kb) [file 11302_2015_9449_Fig7_ESM.gif]
